# SIRT7 Regulates Lipopolysaccharide-Induced Inflammatory Injury by Suppressing the NF-*κ*B Signaling Pathway

**DOI:** 10.1155/2019/3187972

**Published:** 2019-06-11

**Authors:** Kun-Lin Chen, Lian Li, Cheng-Min Li, Yi-Ru Wang, Fang-Xiao Yang, Mei-Qian Kuang, Gen-Lin Wang

**Affiliations:** ^1^College of Animal Science and Technology, Nanjing Agricultural University, Nanjing 210095, China; ^2^Institute of Animal Science, Jiangsu Academy of Agricultural Sciences, Nanjing 210014, China

## Abstract

Mastitis has severely affected the cattle industry worldwide and has resulted in decreased dairy production and cattle reproduction. Although prevention and treatment methods have been implemented for decades, cattle mastitis is still an intractable disease. Sirtuin 7 (SIRT7) is an NAD^+^-dependent deacetylase that is involved in various biological processes, including ribosomal RNA synthesis and protein synthesis, DNA damage response, metabolism, and tumorigenesis. However, whether SIRT7 participates in inflammation remains unknown. Our results revealed that SIRT7 is downregulated in tissue samples from mastitic cattle. Therefore, we isolated dairy cow mammary epithelial cells (DCMECs) from breast tissues and developed an *in vitro* model of lipopolysaccharide- (LPS-) induced inflammation to examine SIRT7 function and its potential role in inflammation. We showed that SIRT7 was significantly downregulated in LPS-treated DCMECs. SIRT7 knockdown significantly increased the LPS-stimulated production of inflammatory mediators, like reactive oxygen and nitric oxide, and upregulated TAB1 and TLR4. In addition, SIRT7 knockdown significantly increased the phosphorylation of TAK1 and NF-*κ*Bp65 in LPS-treated DCMECs. Moreover, SIRT7 knockdown promoted the translocation of NF-*κ*Bp-p65 to the cell nucleus and then increased the secretion of inflammatory cytokines (IL-1*β* and IL-6). In contrast, SIRT7 overexpression had the opposite effects when compared to SIRT7 knockdown in LPS-treated DCMECs. In addition, SIRT7 overexpression attenuated LPS-induced DCMEC apoptosis. Taken together, our results indicate that SIRT7 can suppress LPS-induced inflammation and apoptosis via the NF-*κ*B signaling pathway. Therefore, SIRT7 may be considered as a potential pharmacological target for clinical mastitis therapy.

## 1. Introduction

Clinical mastitis (CM) is a widespread and economically important disease that affects dairy cattle by reducing milk quality and production. In addition, CM increases the risk of culling and death, and treatment is costly [1, 2]. Approximately 80% of all intramammary *Escherichia coli* (*E. coli*) infections result in CM [3–5]. The major outer membrane component of *E. coli* is lipopolysaccharide (LPS), which activates the TLR4-NF-*κ*B signaling pathway, stimulating the release of inflammatory cytokines [6]. In turn, these cytokines and mediators further aggravate the pathology and inflammatory responses involved in mastitis.

Mastitis is characterized by increased secretion of proinflammatory cytokines, such as TNF-*α*, IL-1*β*, and IL-6, which have been demonstrated to direct the migration of neutrophils to the site of infection [7]. Furthermore, exposing neutrophils and macrophages to LPS rapidly induces the secretion of many mediators, such as nitric oxide (NO), prostaglandin E2 (PGE2), and reactive oxygen species (ROS) [8]. NO is a major inflammatory mediator that is involved in various physiological processes including vasodilatation and the increase in vascular permeability [9]. One study showed that proinflammatory cytokines or LPS can induce the expression of NO synthase (iNOS), which further promotes NO synthesis [10]. ROS act as secondary messengers and participate in cell growth, adhesion, differentiation, senescence, and apoptosis, as well as the modification of various signaling molecules [11, 12]. Excessive production and accumulation of ROS are detrimental to cells and tissues. An unbalanced redox state is a key step in the development of various inflammatory diseases. Thus, inhibition of NO and ROS production is a promising approach in the treatment of inflammatory diseases. The transcription factor NF-*κ*B is a pleiotropic regulator of many genes and is involved in physiological and pathological processes, including immunity, inflammation, and metabolism [13]. NF-*κ*B normally binds to inhibitor of *κ*B*α* (I*κ*B*α*) and assumes an inactive state in the cytosol. By contrast, phosphorylation and degradation of I*κ*B*α* cause the release and subsequent translocation of NF-*κ*B to the nucleus, thereby activating the expression of downstream target genes [14]. TAB1 is a TAK1-interacting protein that promotes TAK1 kinase activity via the autophosphorylation of key serine/threonine sites in the kinase activation loop [15]. TAB1 forms a complex with TAK1, which acts as a key component of LPS-mediated IKK-NF-*κ*B upstream signaling, and is involved in regulating the immune response.

Sirtuin7 (SIRT7) is a nicotinamide adenine dinucleotide oxidized form- (NAD^+^-) dependent deacetylase. SIRT7 is implicated in various processes, such as aging, DNA damage repair, and cell signaling transduction [16, 17]. In addition, SIRT7 has been shown to function in aging-related processes. SIRT7^−/−^ mice had shorter lifespans, showed decreased stress resistance, and developed age-dependent inflammatory cardiomyopathy, which implies that SIRT7 is involved in inflammatory processes [18]. Currently, treatment and control of CM and mastitis are primarily based on the use of antimicrobial drugs [19]. Although antibiotic treatments have achieved remarkable efficiency against mastitis, antibiotic residues found in milk and meat are harmful to human health [12]. In recent years, numerous nonantimicrobial drugs and treatment strategies against CM have been reported. Despite decades of intensive research and implementation of preventive measures, mastitis remains an intractable disease. Considering the potent effects of SIRT7 and its function in inflammation, we hypothesized that SIRT7 might be involved in mastitis. Therefore, in the present study, we utilized a dairy cow mammary epithelial cell (DCMEC) model of LPS-induced mastitis to investigate the function of SIRT7 in the induction and progression of the inflammatory response.

## 2. Materials and Methods

### 2.1. Collection of Mammary Tissues

The protocol of the present study was approved by the Institutional Animal Care and Use Committee of Nanjing Agricultural University. We collected five normal and five inflammatory mammary tissue samples from five Chinese Holstein cows in a local slaughterhouse. The samples were immediately frozen in liquid nitrogen and stored until analysis.

### 2.2. Cell Culture and LPS Treatment

*In vitro* culture of DCMECs was conducted as follows: Breast tissues were cut into 1.0 mm × 1.0 mm × 1.0 mm pieces and washed five times in PBS. Next, to the samples, we added an enzyme mixture (1.5 g/L type I collagenase, 1.5 g/L type II collagenase, and 1.5 g/L trypsin) (Sigma-Aldrich, Cat: C0130 and C6885) and incubated samples at 37°C at 100 r/min in an oscillation incubator for 3 h. Samples were then filtered through a 100-mesh sieve and centrifuged at 130 × g for 5 min. The supernatants were discarded, and then cells were cultured in Dulbecco's modified Eagle's medium (DMEM) supplemented with 10% fetal bovine serum (Gibco, Cat: 10438026) and 1% antibiotic-antimycotic solution in a humidified incubator with 5% CO_2_ at 37°C. After 1 h, the supernatants were collected and subcultured in new flask bottles in order to discard unwanted cells. DCMECs were identified by anti-Cytokeratin 18 antibody (Abcam, Cat: ab52459, 1 : 100). Confirmed DCMECs were treated with LPS.

### 2.3. Transfection

DCMECs were seeded in a six-well plate and cultured for 24 h until they reached 50%-60% confluence. DCMECs were transfected with small interfering RNA (siRNA) or plasmids using Lipofectamine 2000 (Invitrogen, Carlsbad, Cat: 11668027). Experimental siRNA oligos or nontargeting control siRNAs were transfected in 100 pmol amounts. Cells were transfected with 3 *μ*g of pcDNA3.0-SIRT7 for SIRT7 overexpression or an empty vector (control plasmid pcDNA3.0). Transfection efficiency was determined via quantitative reverse-transcription PCR (qRT-PCR) and western blotting. The sequences of the SIRT7 and negative control siRNAs, which were synthesized by Shanghai GenePharma Co. Ltd., are listed in Table 1.

### 2.4. Total RNA Extraction and qRT-PCR

Total RNA was extracted with the Trizol Reagent (Invitrogen, Cat: 15596026) at 48 h after siRNA transfection. Reverse transcription was performed using PrimeScript™ RT Master Mix (TaKaRa, Cat: RR036A). Expression levels of mRNA were quantified via real-time PCR. Expression levels of all target genes were normalized to those of endogenous reference gene *β*-actin, according to an optimized comparative Ct (2^−ΔΔCt^) value method, where ΔΔ = ΔCt_target_ − ΔCt_*β*−actin_. Primer sequences are listed in Table 2.

### 2.5. Western Blot Analysis

To extract total proteins, cells were incubated with lysis buffer and centrifuged at 15000 × g for 20 min at 4°C, and the supernatant was collected. Nuclear and cytoplasmic proteins were isolated using an extraction kit (Beyotime, Cat: P0027) following the manufacturer's instructions. Proteins were separated via SDS-PAGE and transferred onto polyvinylidene difluoride (PVDF, Life Technologies, Cat: LC2002) membranes. Next, membranes were blocked in TBST (TBS containing 0.1% Tween 20) containing 5% nonfat milk for 1 h, followed by incubation at 4°C overnight with primary antibodies. Protein bands were incubated with the secondary antibodies for 1 h at room temperature. Finally, the membranes were visualized with an enhanced chemiluminescence detection system (Amersham Biosciences Corp., Piscataway, NJ). Densitometry analysis was performed using ImageJ software (National Institutes of Health, Bethesda, MD, USA). The signal intensity for each protein band was normalized against the *α*-tubulin loading control. The following commercially available antibodies were used: anti-SIRT7 (Proteintech, Cat: 12994-1-AP, 1 : 2000), anti-TAB1 (Proteintech, Cat: 14819-1-AP, 1 : 1000), anti-p-TAK1 (Bioss, Cat: bs-5435R, 1 : 1000), anti-IL-1*β* (ABclonal Technology, Cat: A1112, 1 : 2000), anti-IL-6 (Abbexa, Cat: abx015895, 1 : 2000), anti-TLR4 (Abcam, Cat: ab22048, 1 : 1000), anti-GAPDH (Proteintech, Cat: 10494-1-AP, 1 : 4000), anti-Histone-H3.1 (Beyotime, Cat: AF0009, 1 : 500), anti-NF-*κ*Bp65 (CST, Cat: 6956, 1 : 2000), and anti-phospho-NF-*κ*Bp65 (CST, Cat: 3033, 1 : 2000).

### 2.6. ROS and NO Staining Assay

Reactive oxygen species (ROS) and nitric oxide (NO) levels were assessed using assay kits based on DCFH-DA (Beyotime, Cat: S0033) and DAF-FM DA (Beyotime, Cat: S0019), respectively. Briefly, DCMECs were seeded in 24-well plates and incubated with 10 *μ*M DCFH-DA and 2 *μ*M DAF-FM DA reaction mixtures for 30 min at 37°C. Then, the cells were quickly washed twice (quickly) with PBS and visualized under a confocal laser-scanning microscope (Zeiss LSM 700 META). The percentages of ROS- or NO-positive cells were determined based on counts from five randomly selected visual fields.

### 2.7. Immunofluorescence Staining

The cells were washed three times with PBS (37°C), and fixed with 4% paraformaldehyde (in PBS) at room temperature for 30 min. Then, cells were permeabilized with 0.5% Triton X-100 in PBS for 20 min. After 1 h in blocking buffer (1% BSA-supplemented PBS), the cells were incubated at 4°C overnight with mouse anti-cytokeratin-18 (Abcam, Cat: ab52459, 1 : 100) or rabbit anti-NF-*κ*Bp-p65 (1 : 100). After three 5 min washes in PBS, the cells were labeled with a fluorescein isothiocyanate- (FITC-) conjugated goat anti-mouse IgG (H+L) antibody or a tetramethylrhodamine- (TRITC-) conjugated goat anti-rabbit IgG (H+L) antibody at room temperature for 1 h. The cells were stained with Hoechst 33342 for 10 min and then mounted on glass slides. After three washes with PBS, cells were imaged under a confocal microscope (Zeiss LSM 700 META).

### 2.8. Enzyme-Linked Immunosorbent Assay (ELISA) IL-6 and IL-1*β* Levels

The levels of IL-6 and IL-1*β* in the treated cell medium were determined according to the instructions for the IL-6 (Shanghai Lengton Bioscience Co. Ltd., Cat: BPE92153) and IL-1*β* (Shanghai Lengton Bioscience Co. Ltd., Cat: BPE92157) ELISA kits. Finally, the absorbance of each well was measured at 450 nm.

### 2.9. Flow-Cytometer Detection of Apoptosis

Cell apoptosis was detected with an Annexin-V/PI kit (BD Biosciences, Cat: 556547). After transfection, the cells were incubated with Annexin-V/PI at room temperature for 25 min. Then, the apoptotic cells were quantified with a FACSCalibur flow cytometer (FCM) (BD Biosciences, Bedford, MA, USA). The data were analyzed by FlowJo software.

### 2.10. Statistical Analysis

All data are expressed as mean ± SEM. Differences between groups were subjected to analysis of variance (ANOVA) or the *t*-test. Data with a *P* value less than 0.05 were considered statistically significant. All the statistical analyses were performed in GraphPad Prism 6.01 software (GraphPad Software Inc., San Diego, CA).

## 3. Results

### 3.1. SIRT7 Expression in Tissue Samples from Mastitis Cattle and DCMEC Isolation

**Figure 1:**
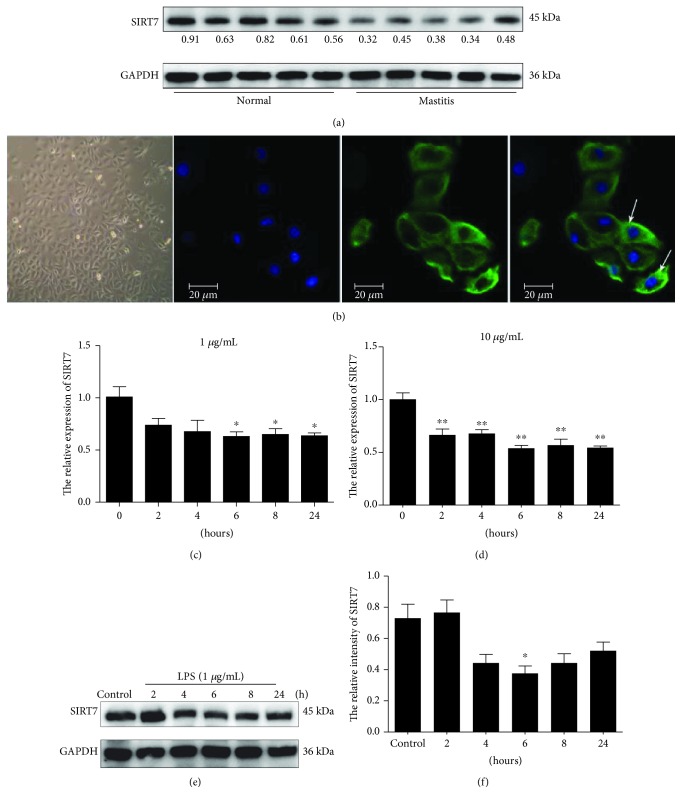
SIRT7 expression in tissues from mastitic cattle and LPS-treated DCMECs. (a) SIRT7 expression was reduced in five different samples from mastitic cattle. (b) DCMECs were cultured *in vitro* and identified using antibodies specific to cytokeratin-18 via immunofluorescence staining. The white arrows indicate the specific localization of cytokeratin-18. Blue: DNA; green: cytokeratin. (c, d) qRT-PCR was carried out to evaluate SIRT7 expression in DCMECs treated with varying concentrations of LPS (1 *μ*g/mL LPS in (c); 10 *μ*g/mL LPS in (d)) at different time points. (e) SIRT7 expression in DCMECs at different time points was evaluated via western blotting after LPS (1 *μ*g/mL LPS) treatment of DCMECs. (f) Quantification of SIRT7 protein levels in DCMECs using ImageJ after LPS (1 *μ*g/mL LPS) treatment of DCMECs. Data are expressed as mean ± S.E.M.^∗^*p* < 0.05 and ^∗∗^*p* < 0.01.

SIRT7 was significantly downregulated in the five tissue samples from mastitis cattle as compared to that in normal samples (Figure 1(a)). This finding indicated that SIRT7 is potentially involved in the pathogenesis of CM. Based on immunofluorescent staining (Figure 1(b)), we concluded that the cells were epithelial cells and were thus used for the subsequent experiments.

### 3.2. SIRT7 is Downregulated in LPS-Treated DCMECs, Which Showed Inflammatory Characteristics

SIRT7 mRNA and protein expression were significantly downregulated in LPS-treated DCMECs relative to that in control cells after 6 h treatment (Figures 1(c)–1(f)). The *in vitro* LPS-treated cell culture model showed a decrease in SIRT7, similar to that in cattle mastitis.

### 3.3. SIRT7 Inhibits LPS-Induced ROS and NO Production

**Figure 2:**
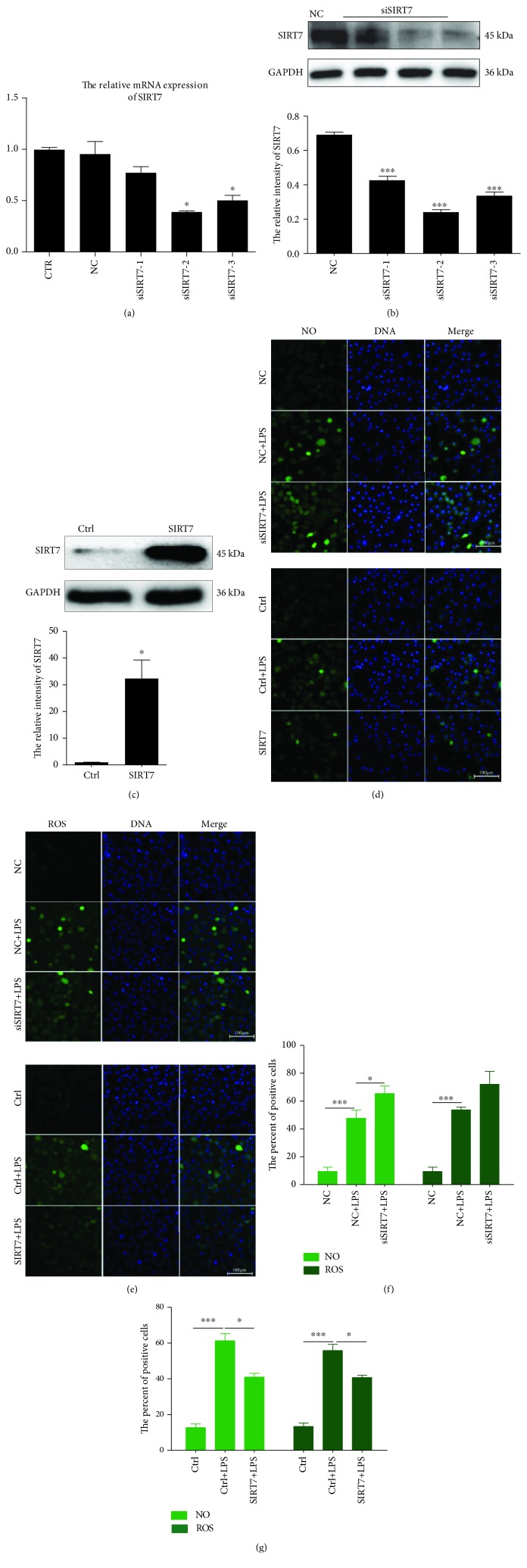
SIRT7 regulated NO and ROS production in LPS-treated DCMECs. (a, b) DCMECs were transfected with three different SIRT7-targeted siRNAs. Knockdown efficiency was assessed by qRT-PCR and western blotting. (c) pcDNA3.0-SIRT7 was used for SIRT7 overexpression. Overexpression efficiency was assessed via western blotting. (d) NO was detected with DAF-FM DA. Scale bar: 100 *μ*m; green: DAF-FM DA; blue: DNA. (e) ROS was labeled with DCFH-DA. Scale bar: 100 *μ*m; green: DCFH-DA; blue: DNA. (f, g) Percentages of NO- and ROS-positive cells were calculated from the counts in five randomly selected visual fields. CTR cells are not transfected; NC (negative control) means the cells were transfected with nontargeting control siRNA; Ctrl (Control) means the cells were transfected with empty vector pcDNA3.0; SIRT7 cells were transfected with pcDNA 3.0-SIRT7. Data are expressed as mean ± S.E.M.^∗^*p* < 0.05 and ^∗∗∗^*p* < 0.001.

To evaluate the biological role of SIRT7 in the induction and progression of the inflammatory response, three different small interfering RNAs (siRNAs) targeting SIRT7 were designed and transfected into DCMECs. The levels of SIRT7 mRNA and protein were found to be significantly downregulated after siRNA transfection (Figures 2(a) and 2(b)). Of the evaluated siRNAs, transfection with siSIRT7-2 resulted in the most efficient knockdown of SIRT7. Therefore, siSIRT7-2 was selected for the subsequent experiments. Meanwhile, in overexpression experiments, SIRT7 was significantly upregulated in cells transfected with pcDNA3.0-SIRT7 compared to that in cells in the control group (Figure 2(c)).

After LPS treatment, NO levels significantly increased compared to that in the control group (Figure 2(d)). SIRT7 knockdown promoted LPS-induced NO production, whereas SIRT7 overexpression significantly attenuated LPS-induced NO generation.

In addition, SIRT7 is also involved in LPS-induced ROS production (Figure 2(e)). The results revealed that LPS treatment induced ROS production in DCMECs. SIRT7 knockdown significantly increased ROS production, while SIRT7 overexpression significantly decreased ROS production (Figure 2(e)). We randomly selected five fields of view to quantify the number of NO- and ROS-positive cells after the SIRT7 knockdown or overexpression in DCMECs, and the results were consistent with our initial observations (Figures 2(f) and 2(g)). Therefore, our data indicated that SIRT7 inhibited LPS-induced NO and ROS production in DCMECs.

### 3.4. SIRT7 is Involved in NF-*κ*B Signaling

**Figure 3:**
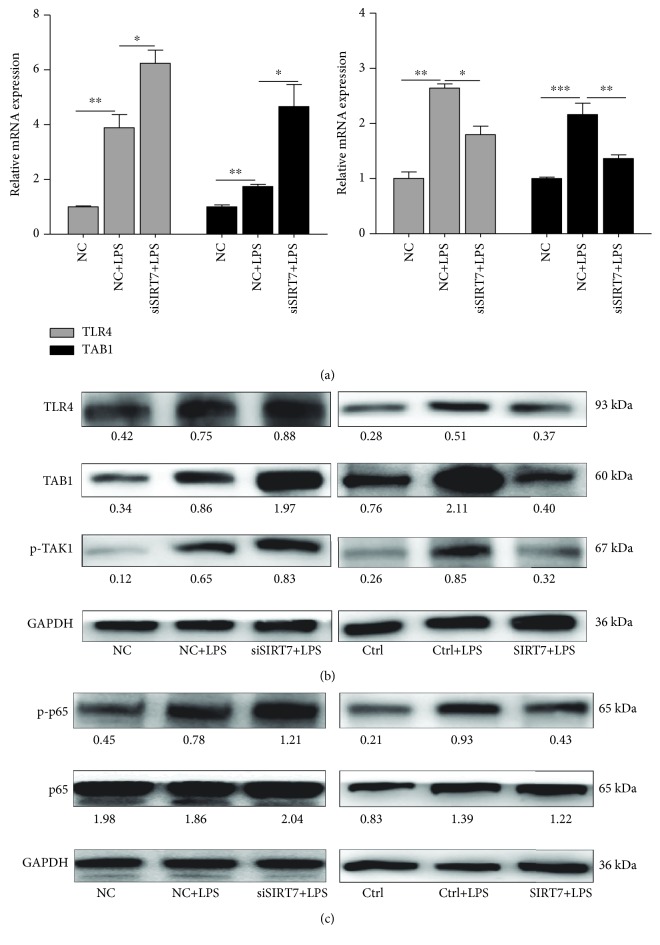
SIRT7 is involved in NF-*κ*B signaling. (a) Relative mRNA expression levels of TLR4 and TAB1 after SIRT7 knockdown or overexpression in LPS-treated DCMECs were measured via qRT-PCR. (b, c) TLR4 and TAB1 levels and the phosphorylation levels of TAK1 and NF-*κ*Bp65 after the SIRT7 knockdown or overexpression in LPS-treated DCMECs were measured via western blotting. Data are expressed as mean ± S.E.M.^∗^*p* < 0.05, ^∗∗^*p* < 0.01, and ^∗∗∗^*p* < 0.001.

Our results showed that LPS treatment increases mRNA and protein expression of TLR4 and TAB1. SIRT7 knockdown with LPS treatment further increased mRNA and protein levels of TLR4 and TAB1. Consistently, SIRT7 overexpression significantly attenuated the LPS-induced increase in TLR4 and TAB1 mRNA and protein levels (Figures 3(a) and 3(b)). Additionally, TAK1 phosphorylation was increased in LPS-treated DCMECs. NF-*κ*Bp65 is a subunit of NF-*κ*B and is essential for the regulation of immune responses. Our data suggested that SIRT7 knockdown significantly increased LPS-induced NF-*κ*Bp65 phosphorylation, whereas SIRT7 overexpression blocked NF-*κ*Bp65 phosphorylation in LPS-treated DCMECs (Figure 3(c)).

### 3.5. SIRT7 Regulates Nucleocytoplasmic Translocation of NF-*κ*Bp65

**Figure 4:**
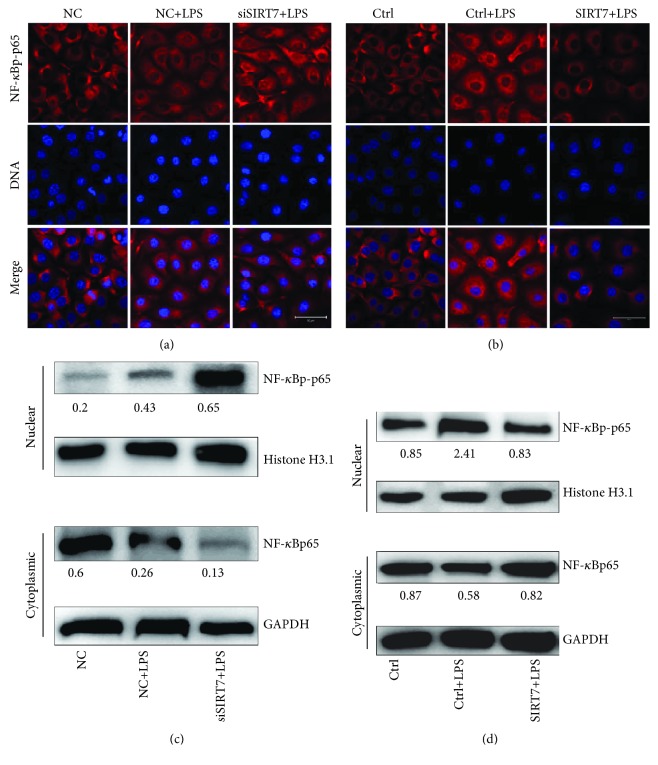
SIRT7 regulates nucleocytoplasmic translocation of NF-*κ*Bp-p65. (a, b) DCMECs were transfected with siSIRT7 or SIRT7 plasmid for 24 h and treated with LPS for 6 h. Immunofluorescence staining was conducted with an anti-NF-*κ*Bp-p65 antibody. Red: NF-*κ*Bp-p65; blue: DNA; scale bar: 50 *μ*m. (c) The protein levels of NF-*κ*Bp-p65 in the nucleus and NF-*κ*Bp65 in the cytoplasm were analyzed via western blotting after the cells were transfected with siSIRT7 or SIRT7 plasmid.

After LPS stimulation in DCMECs, NF-*κ*Bp65 was phosphorylated and underwent nuclear translocation (Figures 4(a) and 4(b)). SIRT7 knockdown promoted nuclear translocation of NF-*κ*Bp-p65, whereas SIRT7 overexpression inhibited this process in LPS-treated DCMECs. Western blotting data confirmed that NF-*κ*Bp-p65 levels in the nuclei of LPS-treated DCMECs increased after SIRT7 depletion, but decreased after SIRT7 overexpression. In contrast, NF-*κ*Bp65 levels were reduced in the cytoplasm after SIRT7 depletion, but increased following SIRT7 overexpression (Figures 4(c) and 4(d)).

### 3.6. Effects of SIRT7 on the Secretion of Proinflammatory Cytokines

**Figure 5:**
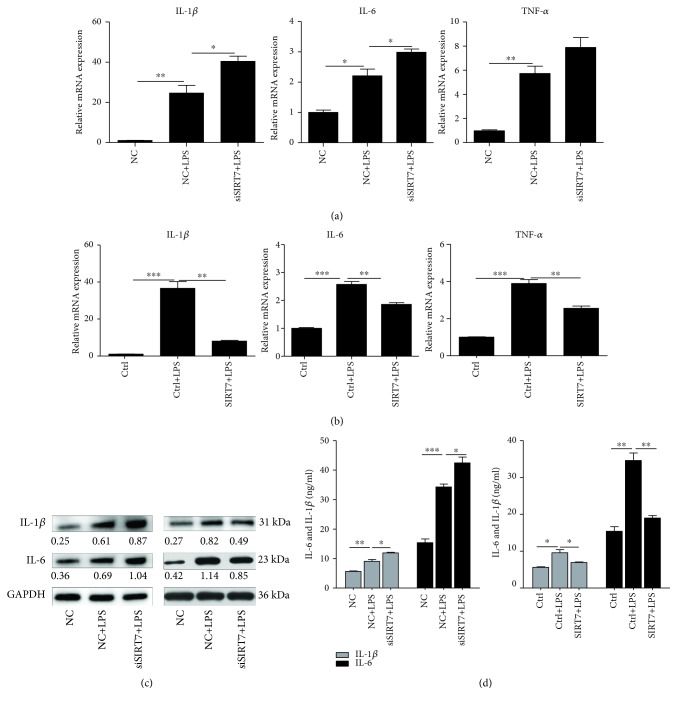
SIRT7 regulates proinflammatory cytokines in LPS-treated DCMECs. (a, b) After transfection for 48 h, DCMECs were treated with LPS for 6 h. The levels of TNF-*α*, IL-1*β*, and IL-6 mRNA were determined by qRT-PCR. (c) The protein levels of IL-1*β* and IL-6 were determined via western blotting. (d) The levels of IL-1*β* and IL-6 in the cell medium were detected by ELISA. Data are expressed as mean ± S.E.M.^∗^*p* < 0.05, ^∗∗^*p* < 0.01, and ^∗∗∗^*p* < 0.001.

Our data showed that SIRT7 depletion significantly increased the mRNA expression of IL-1*β* and IL-6 (Figure 5(a)), whereas SIRT7 overexpression significantly inhibited the expression of IL-1*β*, IL-6, and TNF-*α* (Figure 5(b)). Changes in IL-1*β* protein levels were also consistent with the observed changes in mRNA levels after SIRT7 depletion or overexpression in LPS-treated DCMECs (Figure 5(c)). And ELISA results also showed the same changes on the secretion of proinflammatory cytokines IL-1*β* and IL-6 in the cell medium (Figure 5(d)).

### 3.7. SIRT7 Depletion Increases LPS-Induced DCMEC Apoptosis

**Figure 6:**
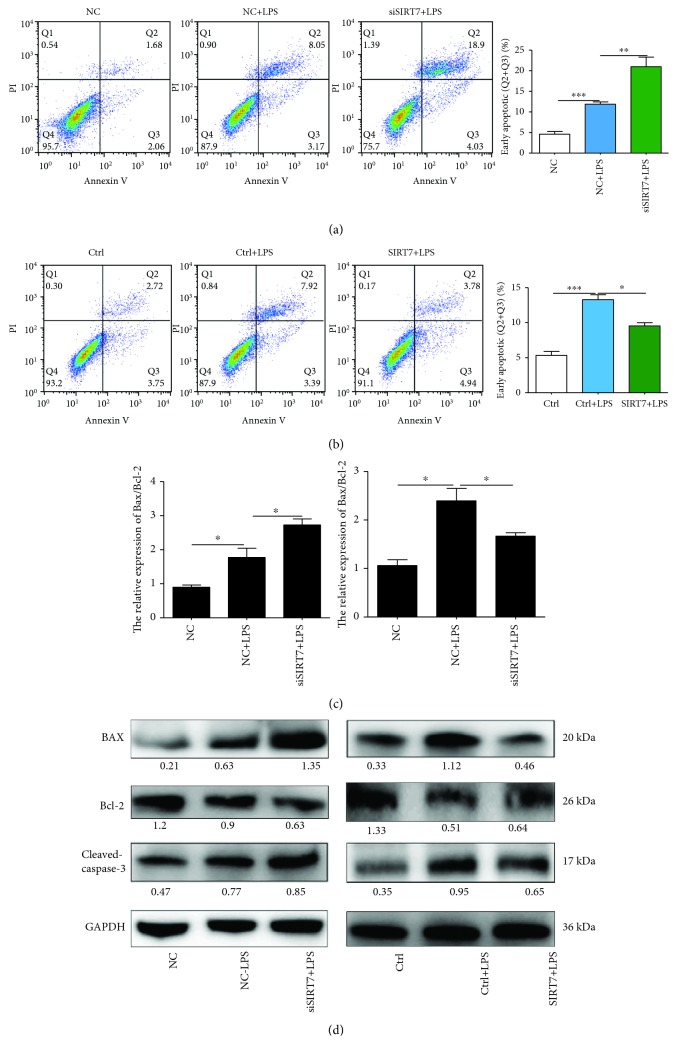
SIRT7 alleviates LPS-induced apoptosis in DCMECs. (a, b) DCMECs were transfected with siSIRT7 or pcDNA3.0-SIRT7 for 48 h and then treated with LPS for 6 h. The percentages of apoptotic cells were examined by flow cytometry. (c) The levels of Bax and Bcl-2 mRNA were determined via qRT-PCR. (d) The protein levels of Bax, Bcl-2, and cleaved-caspase-3 were determined by western blotting. Quantification of the relative protein levels are shown under the bands (protein/GAPDH). Data are expressed as mean ± S.E.M.^∗^*p* < 0.05.

After LPS treatment, the percentage of apoptotic cells was significantly increased and depletion of SIRT7 increased LPS-induced DCMEC apoptosis (Figures 6(a) and 6(b)). In contrast, when we overexpressed SIRT7, LPS-induced apoptosis of DCMECs was significantly attenuated. To further explore the effects of SIRT7 on LPS-induced DCMEC apoptosis, qRT-PCR and western blotting were used to measure the protein and mRNA expression levels of apoptosis-related genes. SIRT7 knockdown significantly increased the Bax/Bcl-2 ratio in LPS-treated DCMECs at both mRNA and protein levels (Figures 6(c) and 6(d)). As expected, overexpression of SIRT7 significantly decreased the Bax/Bcl-2 mRNA ratio and the protein levels of Bax. Furthermore, we found that overexpression of SIRT7 decreased the expression of cleaved-caspase-3. Taken together, these findings indicated that SIRT7 is also involved in LPS-induced DCMEC apoptosis.

## 4. Discussion

SIRT7 is a NAD^+^-dependent deacetylase and is the only member of the sirtuin family that localizes to the nucleolus [20]. We have previously shown that SIRT7 participates in breast cancer cell proliferation, migration, and tumor progression by activating p38MAPK [21]. However, whether SIRT7 is involved in the induction and progression of the inflammatory response, especially in cattle mastitis, remains unknown. In the present study, SIRT7 was downregulated in tissues from cattle with CM and in LPS-treated DCMECs, in agreement with another study that reported downregulated SIRT7 in LPS-treated THP-1 cells [22]. It has also been shown that SIRT7 expression is reduced in response to H_2_O_2_-induced oxidative stress in H9C2 cells [23]. However, the mechanism of SIRT7 downregulation under stress conditions needs further investigation, which could be related to other SIRT family members.

Various studies have shown that LPS stimulation induces the secretion of various proinflammatory mediators, such as NO and ROS [24, 25]. The production and secretion of NO can induce local tissue damage and further trigger inflammation [26]. Therefore, the inhibition of NO production is an important therapeutic strategy for the treatment of inflammatory diseases [27]. ROS, another proinflammatory mediator, also plays crucial roles in the pathogenesis of various inflammatory disorders [28]. In some inflammatory-related lung diseases, activated inflammatory cells like neutrophils and macrophages are thought to release considerable amounts of ROS, which in turn promote inflammation and trigger the development of other related diseases [29]. SIRT7 overexpression suppressed NO and ROS secretion in LPS-treated DCMECs, indicating that SIRT7 can suppress the initiation of inflammation by inhibiting NO and ROS production under normal conditions, which is consistent with a previous work [30]. A recent study demonstrated that SIRT7 is also involved in the regulation of mitochondrial homeostasis by deacetylating GABP *β*1, which is important in regulating mitochondrial genes [31]. Various studies have shown that other SIRT family members (SIRT2, SIRT5, and SIRT6) are associated with NF-*κ*B, which plays a critical role in regulating ROS by targeting enzymes which induce ROS, such as NADPH oxidase, inducible NO synthase, cyclooxygenase-2, xanthine oxidoreductase, and cytochrome p450 enzymes [32, 33].

TLR4 is a transmembrane protein that is involved in the innate immune response, which can be activated by LPS. Upon activation, TLR4 stimulates the downstream NF-*κ*B signaling pathways and triggers the production of large amounts of inflammatory cytokines [34, 35]. From previous work, it has been established that transforming growth factor *β*-activated kinase 1 (TAK1) is important for transmitting upstream signals from the receptor complex to the downstream NF-*κ*B signaling pathways. Furthermore, it can be activated by inflammatory mediators, such as IL-1, TNF, and Toll-like receptor ligands [36–38]. As an adaptor of TAK1, TAB1 is constitutively bound to TAK1 and promotes TAK1 autophosphorylation during the inflammatory response [39, 40]. In addition, the TAK1-ECSIT-TRAF6 complex is crucial for the activation of TLR4-mediated NF-*κ*B signaling [41]. Therefore, the TLR4-TAK1-TAB1-mediated NF-*κ*B signaling pathway is essential for inflammation. We found that SIRT7 can attenuate LPS-induced upregulation of TLR4 and TAB1 and can also inhibit TAK1 phosphorylation, indicating that SIRT7 is involved in the TLR4-TAK1-TAB1-mediated NF-*κ*B signaling pathway during cattle mastitis occurrence. Nevertheless, determining how SIRT7 participates in this pathway still requires further exploration.

NF-*κ*B is a key nuclear transcription factor that comprises the p50 and p65 subunits, which associate with inhibitory factor I*κ*B in the cytoplasm of unstimulated cells. In response to various stimuli, NF-*κ*B is activated and induces the degradation and release of I*κ*B*α* from the dimeric complex, followed by the phosphorylation of NF-*κ*Bp65 and its subsequent translocation to the nucleus [42]. Phosphorylation of the NF-*κ*B subunits can either increase or decrease the transcription of the target genes [43] and thus exert a profound effect on NF-*κ*B function. Once NF-*κ*Bp-p65 enters the nucleus, NF-*κ*B initiates the transcription of genes encoding proinflammatory cytokines, including IL-1*β*, IL-6, and TNF-*α* [44]. Our results revealed that SIRT7 also participates in NF-*κ*B phosphorylation and nucleocytoplasmic translocation of NF-*κ*Bp-p65. SIRT7 was concentrated in the nucleus, while p65 is mainly distributed in the cytosol of unstimulated cells. Therefore, SIRT7 could bind to p65 in the nucleus and regulate the translocation of p65 between the nucleus and cytoplasm [45]. However, how SIRT7 regulates p65 translocation needs further investigation. The function of SIRT7 in inflammation is similar to that of the other members of the sirtuin family. For example, SIRT1 can suppress inflammation in multiple tissues and macrophages, and SIRT1 interacts with NF-*κ*Bp65 to inhibit NF-*κ*B-associated transcription [46]. In addition, SIRT2 is a deacetylase of NF-*κ*B, which is important for regulating TNF-*α*-induced NF-*κ*B-dependent gene expression. In unstimulated cells, SIRT2 exits in a complex with p65, but under TNF-*α* stimulation, p65 translocates to the nucleus and is acetylated at K310, K314, and K315 through binding to p300. After stimulation, p65 returned to the cytoplasm and SIRT2 deacetylated p65 [47]. Recent work showed that SIRT7 interacts with SIRT1. Therefore, the function of SIRT7 in NF-*κ*B acetylation could occur through SIRT1 [48]. Depletion of SIRT7 induced a rapid increase in the production of proinflammatory cytokines, including TNF-*α*, IL-1*β*, and IL-6, which supported our hypothesis that SIRT7 is involved in NF-*κ*B-mediated inflammation.

Various studies have shown that increased ROS production and a release of inflammatory cytokines, such as TNF-*α*, IL-6, and IL-1*β*, ultimately lead to apoptotic cell death [49, 50]. The apoptosis-related genes, caspase-3, caspase-8, and Bax, were found to be significantly upregulated in LPS-treated mice [51]. In addition to the function of TLR4 in the LPS-induced inflammatory response, ROS production can trigger the downstream apoptotic pathways, thereby increasing the amounts of Bax, Bcl-2, and cleaved-caspase-3 [52]. Alterations in the expression of both Bcl-2 and Bax are known to trigger the mitochondrial intrinsic apoptosis program and then promote cell apoptosis. One study revealed that SIRT7 promotes gastric cancer growth and inhibits apoptosis of gastric cancer cells by inhibiting miR-34a activity [53]. Our data indicated that SIRT7 could protect cells from the LPS-induced apoptosis by downregulating Bax and cleaved-caspase-3, which is consistent with one study which showed that silencing of SIRT7 decreased antiapoptotic factor Bcl-2 and NF-*κ*B levels [54]. Because the NF-*κ*B signaling pathway is critical in cellular proliferation, apoptosis, and malignant diseases [55], it will be interesting to investigate the regulatory mechanism between SIRT7 and NF-*κ*B.

**Figure 7:**
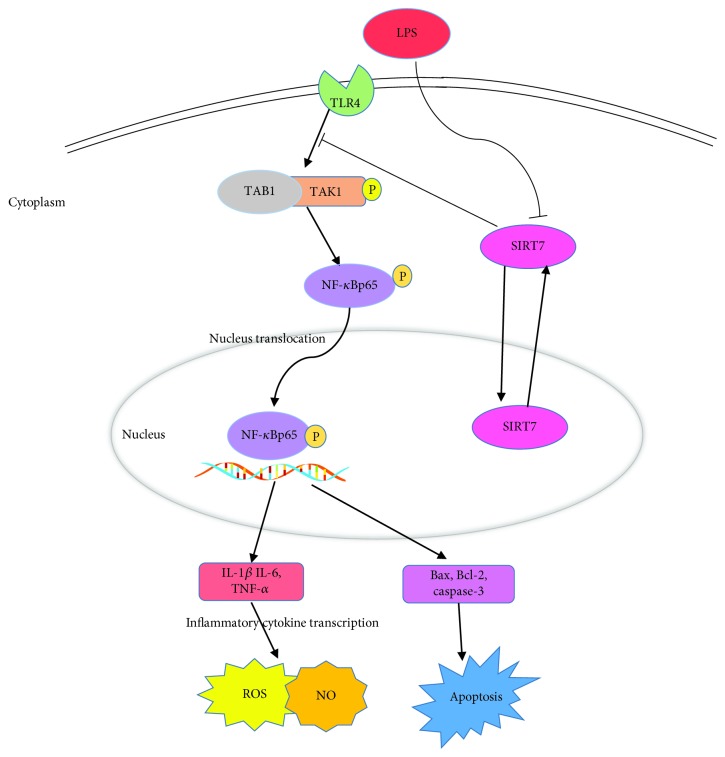
Possible signaling transduction pathways induced by SIRT7 in LPS-treated DCMECs. SIRT7 may attenuate LPS-induced upregulation of TLR4, TAB1, and p-TAK1, deactivate the NF-*κ*B pathway, and downregulate inflammatory cytokines (IL-1*β*, IL-6, and TNF-*α*), ROS, and NO. Subsequently, SIRT7 suppresses apoptosis-related gene expression. As a result, SIRT7 may inhibit the proinflammatory response and initiation of apoptosis in LPS-treated DCMECs.

In conclusion, our results suggest that SIRT7 can suppress LPS-induced inflammation and apoptosis via the NF-*κ*B signaling pathway (Figure 7). Given its anti-inflammatory and antiapoptotic effects, SIRT7 may serve as a potential pharmacological target for CM therapy.

## Figures and Tables

**Table 1:** Primer sequences of siSIRT7.

Genes	Forward	Reverse
siSIRT7-1	5′-GCAGCCUCUAUCCCAGAUUTT-3′	5′-AAUCUGGGAUAGAGGCUGCTT-3′
siSIRT7-2	5′-GCACUCCCAAUAGGGAAUATT-3′	5′-UAUUCCCUAUUGGGAGUGCTT-3′
siSIRT7-3	5′-GCAAGUGUGAUGACGUCAUTT-3′	5′-AUGACGUCAUCACACUUGCTT-3′
Negative control	5′-UUCUCCGAACGUGUCACGUTT-3′	5′-ACGUGACACGUUCGGAGAATT-3′

**Table 2:** Primer sequences of mRNA.

Genes	Forward	Reverse
SIRT7	5′-GAGAGCGAGGACCTGGTGAC-3′	5′-GATAGAGGCTGCCGTGCTGA-3′
TLR4	5′-GGGTTGCTGTTCTCACACTG-3′	5′-AGGTAGCGGAGGTTTCTGAG-3′
IL-1*β*	5′-AGGTGGTGTCGGTCATCGT-3′	5′-GCTCTCTGTCCTGGAGTTTGC-3′
TAB1	5′-GCGATCTCGGCTCCTAGCAA-3′	5′-GCTACTCGGGAGGGCTTAGG-3′
TNF-*α*	5′-ACGGGCTTTACCTCATCTACTC-3′	5′-GCTCTTGATGGCAGACAGG-3′
IL-6	5′-ATGCTTCCAATCTGGGTTC-3′	5′-TGAGGATAATCTGGGTTC-3′
Bax	5′-ATGCGTCCACCAAGAAGC-3′	5′-CCAGTTGAAGTTGCCATCAG-3′
Bcl-2	5′-ATGTGTGTGGAGAGCGTCAA-3′	5′-TCGAAGGAAGTCCAATGTCC-3′
*β*-Actin	5′-TCACCAACTGGGACGACA-3′	5′-GCATACAGGGACAGCACA-3′

## Data Availability

The data used to support the findings of this study are available from the corresponding author upon request.
